# Intravenous ferric derisomaltose for the treatment of iron deficiency anemia

**DOI:** 10.1002/ajh.26124

**Published:** 2021-02-26

**Authors:** Michael Auerbach, David Henry, Thomas G. DeLoughery

**Affiliations:** ^1^ Department of Medicine Georgetown University School of Medicine Washington District of Columbia; ^2^ Department of Medicine, Division of Hematology and Oncology Pennsylvania Hospital Philadelphia Pennsylvania; ^3^ Department of Hematology and Medical Oncology, Knight Cancer Center Oregon Health & Sciences University Oregon Portland

## Abstract

Intravenous (IV) iron is the therapy of choice when oral iron is ineffective or poorly tolerated, yet use has been limited by fears of hypersensitivity reactions (HSRs). Newer formulations that bind iron more tightly and release it more slowly have made the risk of serious or severe HSRs very low. One such formulation, ferric derisomaltose, has been approved in the United States for delivery of 1000 mg iron in a single IV infusion. Ferric derisomaltose rapidly repletes iron parameters with low rates of serious or severe HSRs. Single‐infusion iron repletion offers convenience, eliminates adherence concerns, and reduces healthcare resource utilization.

## 1 HISTORY OF THE DEVELOPMENT OF FERRIC DERISOMALTOSE

After the first parenteral iron formulation, ferric oxyhydroxide, was introduced in 1932,^1^ the perception of toxicity in the medical community was so negative its use was abandoned. Remarkably, in 1952, Baird and Padmore introduced a high molecular weight (HMW) iron dextran (ImFeron, Fisons, Homes Chapel, UK), with a complex carbohydrate core binding elemental iron tightly allowing a larger dose to be administered in a much shorter period of time. Soon after the original approval for intra‐muscular (IM) injection it was shown that the intravenous (IV) route was far less cumbersome, enabled the administration of complete doses in one visit, and was neither less efficacious nor more toxic than IM injections.

While safe and effective, infrequent infusion reactions were reported with HMW iron dextran.^2^, ^3^ Misinterpretations of infusion reactions and misinformation about their cause fomented an ongoing folklore of IV iron danger and resulted in inappropriate interventions with pressers and antihistamines, converting minor, self‐limited reactions into serious adverse events (SAE). HMW iron dextran remained a minor product. In 1989, recombinant human erythropoietin (EPO) was approved for use in dialysis‐associated anemia, yet 3 years later only 60% of patients on dialysis were being treated to target hemoglobin (Hb) concentrations and many not at all. Eschbach and colleagues^4^ later reported marked improvement in EPO responsiveness with the addition of IV iron in the treatment paradigm for anemia chronic kidney disease (CKD) even with iron parameters consistent with iron repletion. The use of HMW iron dextran became standard in the dialysis community with rare SAEs.

In the early 1990s ImFeron was withdrawn from markets worldwide^5^ and Schein Pharmaceuticals in Arizona released Pharmacosmos’ low molecular weight (LMW) iron dextran (INFeD) for use in dialysis, which quickly became the principal parenteral product used in 475 000 patients on dialysis in the US. The SAEs associated with parenteral iron administration were vanishingly rare until 1996, when another HMW iron dextran (Dexferrum, American Regent, Shirley, NY) was released as a less expensive alternative to INFeD. In 1999, during a brief period when LMW iron dextran became unavailable for use in dialysis centers in the US, there was an 11‐fold increase in SAE reports, using FDA's spontaneous adverse reporting system.^6^

Novel parenteral iron formulations followed which supplanted the use of iron dextrans. In 2004, ferric gluconate (FG; Ferrlecit, Watson, Morristown, NJ) demonstrated an exceedingly low rate of infusion reactions in treatment naïve and treatment‐experienced patients^7^ and soon after, iron sucrose (IS; Venofer, American Regent) was approved in the US for iron deficiency anemia associated with CKD. Rapidly, virtually all patients on dialysis in the US were switched to FG and later IS. Also in 2004, Chertow et al.^8^ examining more than 30 million doses of administered IV iron, reported that the overwhelming preponderance of SAEs were due to the HMW formulation, iron dextran (Iron Dextran Injection, Dexferrum). The other formulations were safe with an estimated SAE frequency of <1:250 000 administrations. As a result, Dexferrum has now been removed from markets worldwide and is no longer available.

Low molecular weight iron dextran, despite its excellent safety record,^8^ was removed from the treatment paradigm in most dialysis centers and eventually interest in LMW iron dextran disappeared. The use of IS, administered in 200 mg infusions, was completely reasonable in a patient population with frequent visits to a dialysis center who had no need for complete replacement dosing in a single appointment. For outpatients with iron deficiency due to inflammatory bowel disease, heavy uterine bleeding, pregnancy, cancer, chemotherapy induced anemia, after bariatric surgery, and with other chronic conditions associated with iron lack, the ability to administer a corrective dose of parenteral iron in one or two visits was effectively discarded as a priority and IS became the dominant iron formulation.

Building upon previous experience and a clear unmet need, Pharmacosmos, Vifor Pharma, and AMAG Pharmaceuticals began the development of IV iron formulations with much more complex carbohydrate shells that bound elemental iron much more tightly, released it more slowly, and allowed for complete iron replacement. Ferric carboxymaltose (FCM; Vifor Pharma, Switzerland) was approved in Europe in 2007 and was approved by the FDA in 2013 for administration of up to 1500 mg in two 750 mg infusions 1 week apart. Ferumoxytol (AMAG Pharmaceuticals, USA) was approved by the FDA in 2009 for administration of up to 1020 mg in two 510 mg bolus injections over 17 seconds a minimum of 3 days and up to 8 days apart. Because of fatal and serious infusion reactions, the bolus dosing was changed to infusions over at least 15 min in 2015. Pharmacosmos’ new formulation, initially known as iron isomaltoside 1000 (Monofer, Pharmacosmos, Denmark) but renamed ferric derisomaltose (FDI; Monoferric) following interactions with the FDA, was approved in 22 countries in Europe in 2009. Ferric derisomaltose was approved in Canada (Pfizer) in 2018 and the United States (Pharmacosmos) in January 2020 respectively as Monoferric. Ferric derisomaltose is the only FDA approved formulation allowing a 1000 mg dose in a single infusion.

### 1.1 Carbohydrate structure, labile iron, and parenteral iron toxicity

**FIGURE 1:**
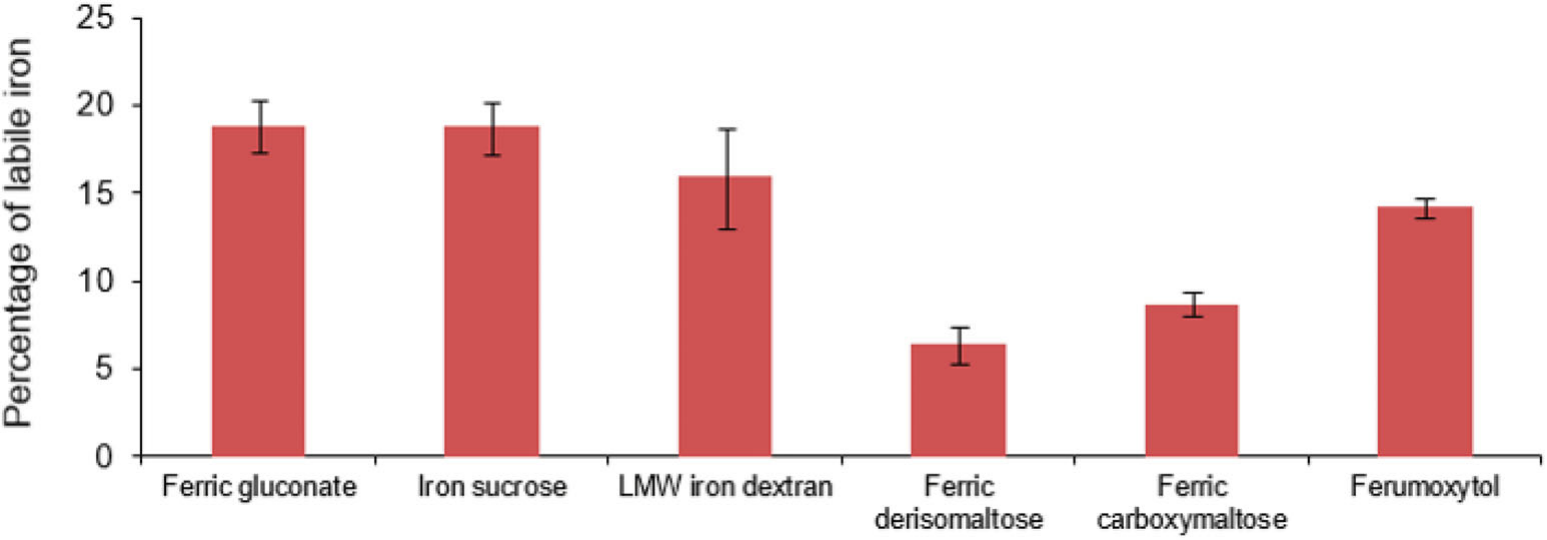
Comparative labile iron pools of parenteral iron products. Labile iron adjusted with the surface/volume ratio of parenteral iron products. The surface to volume ratio is inversely proportional to the iron content. Master data for the figure are published.^9^ Reprinted from *Journal of pharmaceutical and biomedical analysis*, 86, Fütterer S, Andrusenko I, Kolb U, Hofmeister W, Langguth P, Structural characterization of iron oxide/hydroxide nanoparticles in nine different parenteral drugs for the treatment of iron deficiency anaemia by electron diffraction (ED) and X‐ray powder diffraction (XRPD), 2013, with permission from Elsevier [Color figure can be viewed at wileyonlinelibrary.com]

The iron carbohydrate complexes in IS and FG are less stable and release more labile iron necessitating smaller doses to avoid toxicity.^9^ Note, FDI has a short linear structure of linked glucose units that form an iron‐carbohydrate matrix. The matrix structure has high iron stability that allows for rapid infusion (≥ 20 minutes) of a high dose and produces labile iron that represents <1% of the iron dose administered (Figure 1).^10^

**FIGURE 2:**
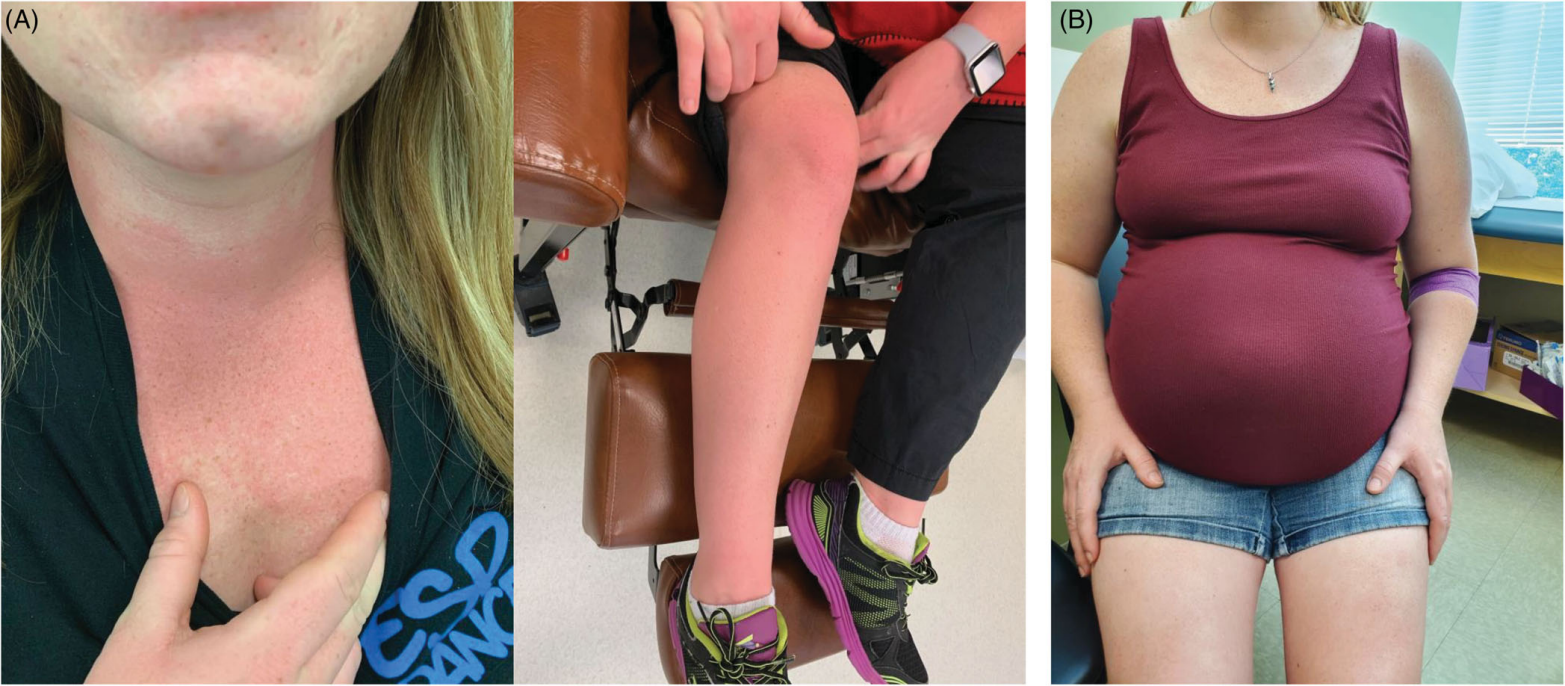
Patient with minor infusion reaction. (A) A typical minor infusion reaction to IV iron with flushing and chest pressure without hypotension, wheezing, stridor or periorbital edema, in 3rd trimester gravida. (B) Photograph taken the next day shows complete resolution of minor reaction, which occurred within 5 min of onset. Premedication with methylprednisolone and famotidine was administered followed by re‐challenge and successful administration of planned dose [Color figure can be viewed at wileyonlinelibrary.com]

Nonetheless, there is a persistent reticence among medical providers to utilize parenteral iron formulations. This is particularly true of those perceived to contain dextrans due to a misunderstood risk of serious or severe hypersensitivity reactions (HSR) despite evidence that classification of IV iron products as dextran‐derived or non‐dextran derived has no clinical relevance.^11^, ^12^ Additionally, based on the preponderance of published evidence, most AEs attributed to parenteral iron are minor self‐limited infusion reactions, due to nontransferrin‐bound labile iron. These reactions usually consist of pressure in the chest or back, and facial flushing or tickling in the throat. These were first described by Fishbane et al,^13^, ^14^ published in a *Lancet* Clinical Update,^15^ and more recently are posited by some to be non‐allergic complement activation‐related (CARPA) reactions.^16^ To the uninitiated or inexperienced they may be misinterpreted as impending anaphylaxis, prompting unnecessary intervention with epinephrine or antihistamines, converting an otherwise minor, self‐limited reaction into a hemodynamically significant SAE, ostensibly due to the intravenous iron. In contradistinction, these minor reactions resolve within minutes and patients may be re‐challenged after symptoms subside with rare re‐appearance of the minor reaction (Figure 2).^11^ Prophylactic medication with methylprednisolone has been shown to mitigate the arthralgia‐myalgia syndrome that may occur following IV iron administration in a double‐blind, randomized trial.^17^ Prospective data supporting the use of steroid before rechallenging patients after a minor infusion reaction are lacking but we use it empirically based on the data above. However, prophylactic antihistamines commonly used in clinical practice can cause somnolence, diaphoresis, tachycardia and hypotension and in prospective studies were reported to be responsible for the majority of adverse events ostensibly associated with the administration of IV iron.^18^ When antihistamines are used to treat minor reactions, they may induce vasoactive reactions treated with vasopressors which exacerbate the minor‐infusion reaction.^16^, ^19^ The subsequent serious or severe HSR is often attributed to the IV iron further propagating the folklore of danger.

## 2 CLINICAL SAFETY AND EFFICACY OF FERRIC DERISOMALTOSE IN US TRIALS

### 2.1 Design of FDI trials

A recent series of clinical trials in the US examined the safety and efficacy of FDI. The FERWON program consisted of two trials; FERWON‐IDA^20^ (ClinicalTrials.gov Identifier: NCT02940886) evaluated the safety and efficacy of high dose FDI in patients with IDA across a broad group of etiologies and FERWON‐NEPHRO^21^ (ClinicalTrials.gov Identifier: NCT02940860) evaluated safety and efficacy of high dose FDI in patients with non‐dialysis‐dependent CKD and IDA. The two trials together included >3000 patients, and a pooled safety analysis was also prespecified.^22^ A co‐primary endpoint in the FERWON trials was the occurrence of serious or severe HSRs and were adequately powered to detect small differences in serious or severe HSRs between FDI and IS, a comparator selected upon recommendation from the FDA because of its low risk for HSRs. Both HSRs and cardiovascular adverse events were adjudicated and confirmed by an independent blinded adjudication committee.^20^, ^21^, ^23^

The design of the FERWON‐IDA^20^ and FERWON‐NEPHRO^21^ trials were closely matched. The trials were prospective, randomized, open‐label, comparative, and multi‐center trials. Eligible patients were randomized 2:1 to receive FDI or IS. So, FDI was administered as a single 1000 mg IV infusion, whereas IS was administered as 200 mg IV injections up to a maximum of five times (a recommended cumulative dose of 1000 mg). In FERWON‐IDA, men or women ≥18 years old with intolerance of, or unresponsiveness to oral iron or Hb at screening sufficiently low to recommend rapid iron store repletion were enrolled. Patients with Hb ≤11 g/dL, transferrin saturation (TSAT) <20%, and *s*‐ferritin <100 ng/mL were included in the trial. The FERWON‐NEPHRO study enrolled adults (≥18 years) with Hb ≤11 g/dL, *s*‐ferritin ≤100 ng/mL (≤300 ng/mL if TSAT ≤30%), CKD defined as either an estimated glomerular filtration rate (eGFR) <60 mL/min/1.73 m^2^ at screening or eGFR <90 mL/min/1.73 m^2^ at screening and kidney damage as indicated by abnormalities in urine composition per medical history and/or intermediate/high risk of cardiovascular disease according to the Framingham model^24^ and either not taking erythropoietin‐stimulating agents or on a stable dose for 4 weeks prior to randomization. Exclusion criteria included iron storage disorders, known hypersensitivity to study drug components or IV iron compounds, IV iron treatment within the 10 days prior to screening, required kidney dialysis, and pregnancy. The co‐primary endpoints of the FERWON trials were the number of adjudicated serious or severe HSRs and change in Hb from baseline to week 8. The FERWON‐EXT 6‐month extension trial enrolled patients from previous randomized controlled trials comparing FDI and IS.^20^, ^21^, ^25^ The primary endpoint was the number of adverse drug reactions after re‐dosing with FDI.^23^

The PHOSPHARE program consisted of two trials (Trial A and B herein) of identical design^26^ (Clinicaltrials.gov identifiers: NCT03238911, NCT03237065). The objective was to compare the development of hypophosphatemia following treatment with FDI or FCM for IDA of mixed etiologies. So, FCM was chosen as the comparator in the PHOSPHARE program due to previous evidence of heightened risk of developing hypophosphatemia during FCM treatment.^27^ The PHOSPHARE trials were open‐label, randomized trials. Eligible patients were randomized 1:1 for treatment with a single IV FDI dose (1000 mg) or IV FCM administered as two 750 mg doses separated by 7 days according to the FDA approved label. Note, PHOSPHARE inclusion and exclusion criteria were consistent with the FERWON‐IDA trial and additionally included exclusions for eGFR <65 mL/min/1.73 m^2^ and serum phosphate level <2.5 mg/dL. The primary endpoint of the PHOSPHARE trials was incidence of hypophosphatemia, defined as serum phosphate <2.0 mg/dL.

One earlier trial of FDI and IS used FDI dosing that is not approved in the US and resulted in higher iron exposure.^25^ To focus on the information most relevant to clinical practice in the US, that trial is not described in detail here.

### 2.2 US trial results

Results for key endpoints are shown in Table 1.

**TABLE 1:** Key results from ferric derisomaltose trials in the US

Parameter	FERWON‐IDA		FERWON‐NEPHRO		PHOSPHARE^a^	
	FDI (N = 1009)	IS (N = 503)	FDI (N = 1027)	IS (N = 511)	FDI (N = 123)	FCM (N = 122)
Serious or severe hypersensitivity reaction, %	0.3	0.4	0.3	0	0.8	1.7
Hypophosphatemia, %	3.9	2.3	3.2	0.8	7.9/8.1	75.0/73.7
Severe hypophosphatemia, %	0	0	0	0	0	11.3
Composite cardiovascular events, %	0.8	1.2	4.1	6.9	NR	NR
Hb change from baseline at week 8, LS Mean g/dL	2.49	2.49	1.22	1.14	NR	NR

Abbreviation: NR, not reported.

^a^ Pooled data from two PHOSPHARE trials except for the primary endpoint, percent with hypophosphatemia, which is shown for trials A and B.

#### 2.2.1 Demographics

In the FERWON‐IDA trial, 989 patients received FDI and 494 IS.^20^ Approximately 90% in each arm were women. And, IDA related to gynecological conditions were approximately 50% of the participant pool, 26% had gastroenterologic conditions. The mean dose (SD) of FDI administered in the single infusion was 975 (145) mg. The majority in the IS arm received five administrations (80%) to a mean (SD) dose of 905 (217) mg. Intravenous FDI was administered to 1019 in the FERWON‐NEPHRO trial, while 506 received IS.^21^ Women comprised 62.5%. Patients with CKD had a comparable mean (SD) eGFR of 35.7 (18.3) mL/min/1.73 m^2^ or 35.2 (18.3) mL/min/1.73 m^2^ in the FDI and IS treatment groups, respectively. The single FDI treatment exposed a mean (SD) iron dose of 993 (71) mg and IS to a cumulative dose of 899 (198) mg over a median of five administrations. A total of 101 patients received a complete dose of FDI in the FERWON‐EXT trial with one receiving a reduced dose due to transient back pain during the infusion.^23^ In the PHOSPHARE trials, 125 received FDI and 117 received FCM across the two identically administered trials.^26^ Greater than 90% enrolled in the PHOSPHARE trials were women and gynecologic bleeding was the most common cause of IDA in the FDI (68%) and FCM (69.2%) arms.

#### 2.2.2 Safety of ferric derisomaltose

The overall incidence of adverse drug reactions (ADRs) in the pooled safety analysis of FERWON trials was similar in the FDI (8.6%) and IS (9.0%) groups (*p* = .68).^22^ Post‐hoc analysis of recurrent ADRs showed that patients treated with FDI experienced fewer days with drug‐related ADRs than those treated with IS (risk ratio 0.67 [95% CI: 0.56; 0.78] *p* < .001).

##### Serious or severe hypersensitivity reactions

In the FERWON trials non‐inferiority of FDI in the adjudicated serious or severe HSR endpoint was met.^20^, ^21^ A serious or severe HSR was observed in 0.3% (95% confidence interval [CI]; 0.06, 0.88) with FDI and 0.4% (95% CI; 0.05, 1.45) with IS in FERWON‐IDA resulting in a non‐significant risk difference of 0.1% (95% CI; −0.91, 0.71). Treatment with FDI resulted in serious or severe HSRs in 0.3% (95% CI; 0.06, 0.86) in FERWON‐NEPHRO (0% in the IS arm) generating a non‐significant risk difference for serious or severe HSRs compared to IS treatment of 0.29% (95% CI; −0.19, 0.77). In the pooled analysis, serious or severe HSRs occurred in 0.3% of the FDI group and 0.2% of the IS group, with a risk difference of 0.10% (95% CI: −0.57; 0.48) confirming non‐inferiority of FDI in the full FERWON population.^22^ No serious adverse drug reactions, including serous or severe HSRs, were reported following FDI re‐dosing in the FERWON‐EXT trial.^23^ In the PHOSPHARE trials, serious or severe HSRs were monitored for AE reporting and occurred in 0.8% with FDI and 1.7% FCM.^26^

##### Hypophosphatemia

Hypophosphatemia has previously been associated with FCM administration.^27^, ^28^ Awareness of hypophosphatemia as an adverse event is important due to the potential for serious musculoskeletal complications including bone loss, osteomalacia, and the potential for severe hypophosphatemia induced by repeated dosing during transient hypophosphatemic periods.^27^ In the FERWON‐IDA trial the hypophosphatemia observed (3.9% with FDI and 2.3% with IS) was transient, with no severe hypophosphatemia (serum phosphate <1.0 mg/dL) linked to either treatment.^20^ Similarly, in the FERWON‐NEPHRO trial few patients treated with FDI (3.2%) and IS (0.8%) developed hypophosphatemia and none developed severe hypophosphatemia.^21^ Re‐dosing in FERWON‐EXT resulted in eight (7.8%) reports of hypophosphatemia with one reported as mild and the others as not clinically significant.^23^

Incidence of hypophosphatemia (serum phosphate <2.0 mg/dL) was the primary endpoint in the PHOSPHARE trials.^26^ Hypophosphatemia was observed in 7.9% treated with FDI in trial A and 8.1% in trial B (75.0% and 73.7% treated with FCM, respectively). Compared to FCM, FDI treatment resulted in a lower rate of treatment associated hypophosphatemia in each trial (trial A: −67.0% [95% CI; −77.4%, −51.5%], trial B: −65.8% [95% CI; −76.6% to −49.8%]). Severe hypophosphatemia did not develop in any treated with FDI. Severe hypophosphatemia developed in 11.3% treated with FCM. At the final study visit on day 35 only one patient (0.9%) treated with FDI across both trials had hypophosphatemia whereas 49 (43%) treated with FCM were hypophosphatemic. In sum, the incidence of hypophosphatemia across the FERWON and PHOSPHARE trials was low with notable differences between the trials likely due to differences in iron deficiency anemia etiology across trials and trial design in PHOSPHARE meant to actively identify hypophosphatemia (i.e., by measuring phosphate at 7 and 14 days after FCM when low phosphate was expected to be most prevalent).

##### Biomarkers of serum phosphate homeostasis

**FIGURE 3:**
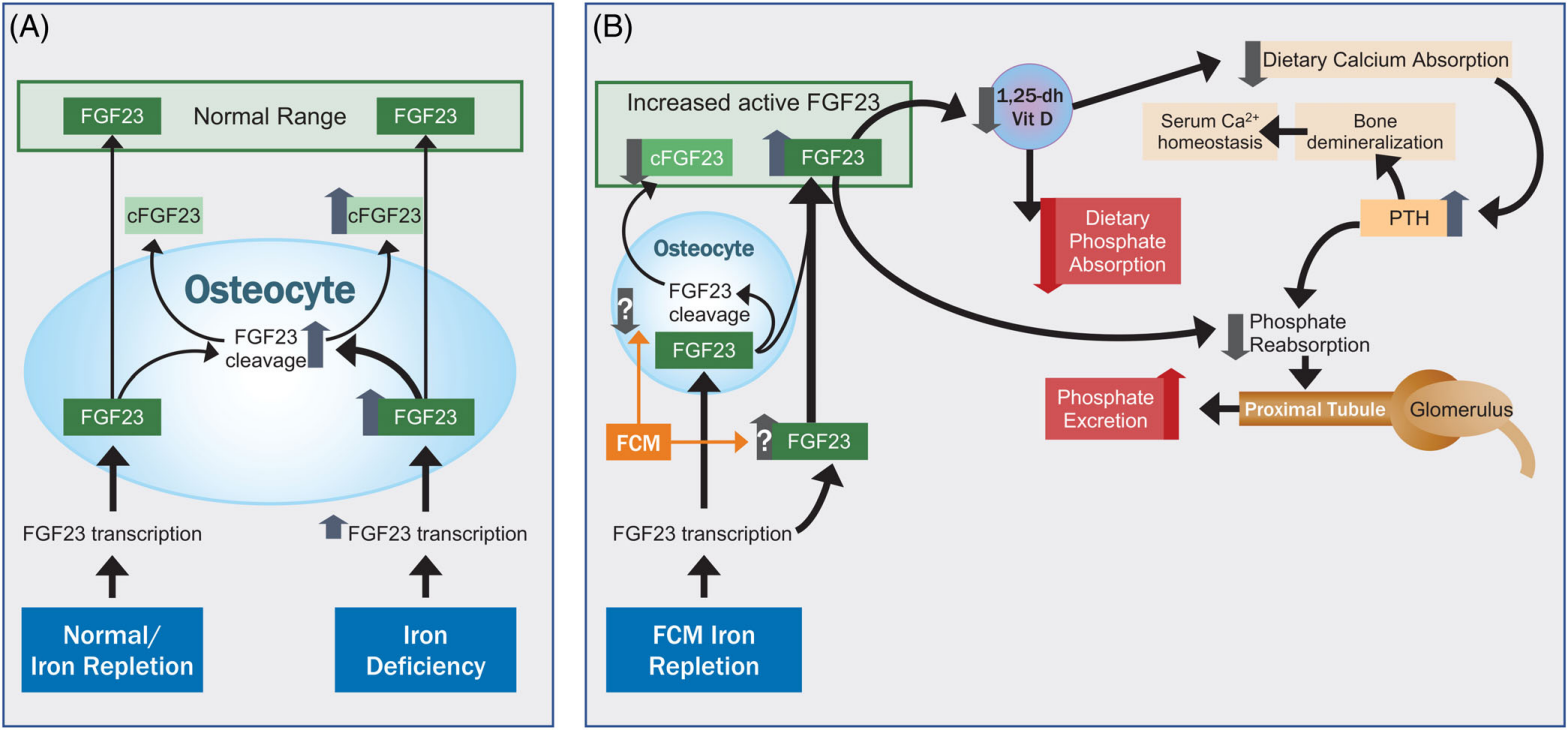
Comparative labile iron pools of parenteral iron products. (A) FGF23 is a circulating hormone that is synthesized primarily in osteocytes and osteoblasts, and is inactivated by cleavage. Under physiologic circumstances and following iron repletion, synthesis and cleavage are coupled to maintain circulating level within a set range. With iron deficiency, *FGF23* gene transcription is increased, but additional synthesis of FGF23 is offset by increased cleavage to maintain normal circulating levels of active FGF23. (B) FCM appears to uncouple the balance between synthesis and inactivation, resulting in increased circulating intact FGF23. The mechanism is unknown, but proposed mechanisms include inhibition of cleavage in osteocytes or activation of FGF23 production in sites without the FGF23 cleavage apparatus. High circulating FGF23 directly inhibits phosphate reabsorption in the proximal tubules of the kidney, and reduces production of serum 1,25‐dihydroxyvitamin D, which in turn reduces dietary phosphate and calcium absorption. Decreased serum calcium increases PTH production to maintain serum calcium homeostasis; PTH also inhibits phosphate reabsorption. The net result is increased phosphate excretion by the kidney and potential for hypophosphatemia. FCM, ferric carboxymaltose; FGF23, fibroblast growth factor‐23; PTH, parathyroid hormone [Color figure can be viewed at wileyonlinelibrary.com]

The PHOSPHARE trials measured biomarkers of serum phosphate homeostasis which support the divergent hypophosphatemia results observed after FDI and FCM treatment.^26^ Mechanistically, intact fibroblast growth factor 23 (iFGF23) causes hypophosphatemia by inducing urinary phosphate excretion and reducing biologically active vitamin D.^28^, ^29^ Reduced biologically active vitamin D limits dietary phosphate and calcium absorption. Reduced serum calcium produces secondary hyperparathyroidism and elevated levels of parathyroid hormone can further promote urinary phosphate excretion (Figure 3). In the PHOSPHARE trials, treatment with FCM resulted in high iFGF23 levels, which were not seen in the FDI arm. Consistent with the mechanistic pathway, urinary phosphate excretion was elevated in the trials’ FCM arm and serum concentrations of biologically active vitamin D were reduced after FCM compared with FDI. Treatment with FDI was associated with near normal serum phosphate levels during the duration of the trial compared with significantly lower phosphate levels with FCM at each time point. Ionized calcium levels were lower with FCM than FDI and consequently, FCM treatment was associated with increased parathyroid hormone levels compared to FDI. After FCM administration elevated parathyroid hormone levels mechanistically support observed increases in urinary phosphate excretion and resultant prolonged hypophosphatemia.

##### Composite cardiovascular events

Evidence from a recent meta‐analysis reported that IV iron improves outcomes in patients with heart failure.^30^ Both CKD and chronic heart failure are often comorbid conditions and each has been reported to incur a greater cost when iron deficiency is present.^31^, ^32^ In the FERWON‐IDA trial the incidence of composite cardiovascular events was consistent across IV iron treatments (0.8% in FDI and 1.2% in IS).^20^ In patients with CKD in the FERWON‐NEPHRO trial, those treated with FDI experienced significantly fewer composite cardiovascular events than those with IS (4.1% vs. 6.9%, respectively [*p* = .025]), and the time to first composite cardiovascular event was significantly longer (*p* = .019) with FDI.^21^ The authors note the difficulty in explaining these differences in the timescale of an 8‐week trial, but suggest that a combination of rapid iron repletion to support mitochondrial function and limited labile iron capable of generating oxidative stress following FDI may provide mechanistic support.^33^ The pooled safety analysis confirmed a significantly lower incidence in the FDI group (63 events in 50 [2.5%] patients) compared with the IS group (48 events in 41 [4.1%] patients; *p* = .018).^22^ In FERWON‐EXT, 6 (5.9%) events were confirmed as cardiovascular events, four events in four with CKD, and none assessed as FDI‐related.^23^ Increased understanding of this important clinical outcome may be generated by the IRONMAN trial, a large trial currently underway that is sufficiently powered to demonstrate reduction of death or worsening heart failure with FDI (ClinicalTrials.gov identifier: NCT02642562).

#### 2.2.3 Efficacy of ferric derisomaltose

##### Hemoglobin response

The FDI treatment in FERWON‐IDA produced a more rapid Hb response than did IS.^20^ A significantly greater increase in Hb was observed from baseline to week 1 and 2 (*p <* .0001). Additionally, the proportion of responders (Hb increase of ≥2 g/dL) was greater with FDI at weeks 1 and 2. By week 4, Hb concentrations were comparable between treatment arms as were the number of responders. At week 8 non‐inferiority of FDI to IS was demonstrated. Similar efficacy results were observed in non‐dialysis dependent patients with CKD in FERWON‐NEPHRO.^21^ The time to achieve an increase in Hb ≥1 g/dL was significantly shorter in the FDI arm (*p =* .017). Significantly faster and more pronounced increases in Hb concentrations were observed after FDI at weeks 1, 2, and 4 (all *p ≤* .021) and FDI resulted in a significantly greater proportion of responders (Hb increase ≥1 g/dL) at these time points. By week 8 Hb concentrations and the proportion of responders were equivalent between treatment arms. In FERWON‐EXT, FDI re‐dosing significantly increased mean Hb concentrations from baseline producing a peak at month three.^23^

##### Fatigue

Fatigue is a common symptom of iron deficiency which negatively impacts quality of life with IDA and reduced fatigue may be an important clinical outcome related to iron repletion.^30^, ^34^ Fatigue was measured in the FERWON trials using the Functional Assessment of Chronic Illness Therapy (FACIT) Fatigue scale.^20^, ^21^ Over half in FERWON‐IDA had severe fatigue at baseline (FACIT‐fatigue score < 30). Both FDI and IS treatment improved fatigue symptoms from baseline by approximately 15 points on the scale. Improvement was more rapid with FDI, which led to a significant difference between treatment groups at week 1 (*p =* .04). More than half in FERWON‐NEPHRO had severe fatigue at baseline, and both groups saw improvement of >10 points in the FACIT‐fatigue score at week 8. Fatigue was not assessed in the PHOSPHARE trials.

#### 2.2.4 Cost

As with the other formulations belonging to the newer generation of IV iron products, FDI is priced higher than the older generation of IV iron products (FG, IS, LMWD). Compared with other newer generation of IV iron products available in the US (Ferumoxitol and FCM), FDI's list price is currently at a premium per treatment course. Actual cost to patients for all IV iron formulations will depend upon insurance coverage and eligibility for patient assistance programs.

## 3 CONCLUSIONS AND FUTURE DIRECTIONS

Oral iron remains frontline therapy for uncomplicated iron deficiency anemia without active bleeding. In situations of intolerance to oral iron, insufficient response to oral iron, IV iron is the preferred route of administration. In practice, the choice of IV iron treatment may reflect concerns about severe HSR reactions attributed to “dextran‐based” IV iron formulations despite clear evidence that the classification has no clinical meaning for risk of HSR reactions.^11^

In 2133 patients administered FDI across the FERWON and PHOSPHARE trials, serious or severe HSRs were observed in only seven patients (0.3%). These results were non‐inferior to IS, an IV iron associated with a low risk for clinically serious or severe HSRs, and similar to FCM. The PHOSPHARE trials demonstrate a low risk of hypophosphatemia with FDI. Low risk for development of hypophosphatemia was also evident in the FERWON trials where the incidence of hypophosphatemia did not reach 4.0%. There were no reported cases of severe hypophosphatemia following FDI treatment and reported hypophosphatemia events were transient. The efficacy of a single 1000 mg dose of FDI in repletion of iron stores is also clear. In each of the trials FDI more rapidly restored iron parameters and improved fatigue compared to IS. These results are consistent with a prior randomized trial comparing FDI to IS that observed more rapid increases in Hb, *s‐*ferritin, and TSAT after FDI treatment.^25^

Single dose iron repletion increases convenience for both providers and patients and decreases the number of intravenous line placements with resultant decreases in infusion reaction and extravasations. Additionally, decreased visits reduce costs for a complete replacement dose and obviate the need for adherence to a treatment plan. The addition of a high‐dose, single visit iron formulation that rapidly repletes iron parameters offers a convenient option for those with iron deficiency anemia, reduced burden on healthcare resources, and an improvement in the treatment paradigm of this common malady.

## CONFLICT OF INTEREST

M.A. reports grants from AMAG Pharmaceuticals and non‐promotional educational talks for Pfizer and Pharmacosmos. T.D. and D.H. have nothing to disclose.
